# Development of a Post-stroke Upper Limb Rehabilitation Wearable Sensor for Use in Sub-Saharan Africa: A Pilot Validation Study

**DOI:** 10.3389/fbioe.2019.00322

**Published:** 2019-11-12

**Authors:** Charmayne M. L. Hughes, Alexander Louie, Selena Sun, Chloe Gordon-Murer, Gashaw Jember Belay, Moges Baye, Xiaorong Zhang

**Affiliations:** ^1^NeuroTech Lab, Health Equity Institute, San Francisco State University, San Francisco, CA, United States; ^2^Department of Kinesiology, San Francisco State University, San Francisco, CA, United States; ^3^School of Engineering, San Francisco State University, San Francisco, CA, United States; ^4^Department of Physiotherapy, University of Gondar, Gondar, Ethiopia

**Keywords:** stroke, kinematics, sensor, rehabilitation, sub-Saharan Africa

## Abstract

The development of context-appropriate sensor technologies could alleviate the significant burden of stroke in Sub-Saharan African rehabilitation clinicians and health care facilities. However, many commercially available wearable sensors are beyond the financial capabilities of the majority of African persons. In this study, we evaluated the concurrent validity of a low-cost wearable sensor (i.e., the outREACH sensor) to measure upper limb movement kinematics of 31 healthy persons, using an 8-camera Vicon motion capture system as the reference standard. The outREACH sensor showed high correlation (*r* range: 0.808–0.990) and agreement (mean difference range: −1.60 to 1.10) with the reference system regardless of task or kinematic parameter. Moreover, Bland-Altman analyses indicated that there were no significant systematic errors present. This study indicates that upper limb movement kinematics can be accurately measured using the outREACH sensor, and have the potential to enhance stroke evaluation and rehabilitation in sub-Saharan Africa.

## Introduction

Stroke is a leading cause of adult long-term disability in sub-Saharan African countries, with a substantial increase in the incidence of stroke in the past 20 years (Owolabi et al., 2015). In contrast to developed countries, stroke occurs 10 to 15 years earlier in sub-Saharan Africa (Murray and Lopez, 1997; Walker et al., 2000, 2003), with stroke survivors exhibiting poorer prognoses (Feigin et al., 2009), and more severe long-term physical disabilities (e.g., weakness or paralysis, sensory loss, spasticity) than their counterparts from developed nations (Owolabi and Ogunniyi, 2009; Bosch et al., 2014). As a result, stroke has a more significant impact on the productive workforce in sub-Saharan Africa than is the case in developed countries (Feigin et al., 2009; Owolabi and Ogunniyi, 2009).

One way to alleviate the burden on rehabilitation clinicians and health care facilities is to develop and utilize context-appropriate technologies in the evaluation and rehabilitation of sub-Saharan African stroke patients. However, in order to ensure sustainability and scalability of said technologies, engineers must consider the extant barriers to healthcare access and infrastructure (e.g., road conditions, internet services, cost of technologies) of each region prior to product development and implementation (Hughes and Ebadat, 2017). For example, Ethiopia is the second most populous country in Africa, with a population of over 105 million inhabitants (World Bank, 2019). Ethiopian stroke rehabilitation services are primarily delivered in urban hospitals, which restricts the ability of the approximately 80% of Ethiopians who live in rural areas (World Bank Group, 2015) to access adequate rehabilitation care. The geographical and cultural context of Ethiopia hinder the use of technological innovations such as rehabilitation robotics and motion capture systems in stroke rehabilitation. However, the scalability and sustainability of technology-aided neurorehabilitation is more possible with inertial measurement units (IMUs), due to their ability to capture detailed upper-limb kinematics, their low cost, and portability (cf. Porciuncula et al., 2018; Walmsley et al., 2018).

There has been a resurgence in the examination of IMUs in clinical care, due in large part to improvements in hardware components and signal processing techniques that can adequately correct sensor bias and dynamic drift (Ricci et al., 2016). For example, van Meulen et al. (2015) examined post-stroke upper limb function during the performance of simulated daily life tasks using 17 MVN Biomech IMUs (Xsens Technologies). High correlation of determination values was obtained when comparing reaching kinematics to the patient's level of arm impairment, suggesting that a body-worn sensor system can accurately and objectively assess arm performance in activities of daily living. Similarly, Carpinella et al. (2014) examined upper limb motor function in individuals with Multiple Sclerosis using a single MTX IMU (Xsens, Netherlands). The authors reported strong significant negative correlations between sensor and clinical test scores, such that lower scores on clinical tests (e.g., Action Research Arm Test, 9 Hole Peg Test) were associated with higher jerk scores and longer task completion times. Taken together, there is growing evidence that IMU sensor systems are capable of accurately measuring upper limb performance in both healthy and impaired populations. Unfortunately, the currently available off-the-shelf sensor platforms (e.g., Xsens, Shimmer, APDM Opal) are beyond the financial capabilities of the majority of Ethiopian persons, require multiple sensors, and require extra programming to generate kinematic performance reports.

With that in mind, the Health Equity Institute at San Francisco State University (USA) and the University of Gondar Comprehensive Specialized Hospital (Ethiopia) have collaborated to develop a low-cost wearable sensor that would provide rehabilitation clinicians with quantitative, yet easily understandable, information about upper limb movements that would enable them to develop patient-specific rehabilitation plans. However, in order to implement the outREACH sensor in Ethiopian stroke care, we first evaluated the concurrent validity of the sensor to measure upper limb movement kinematics, using an eight-camera Vicon motion capture system as the reference standard. To this end, 31 neurologically and physically healthy persons performed tasks commonly used to evaluate post-stroke upper limb function, with movements recorded by both the outREACH sensor and Vicon system.

## Methods

### Participants

A total of 31 adults between 21 and 38 years old (mean age = 24.5, SD = 5.23, 15 men, 16 women) participated in the experiment. Thirty participants were right-handed and one was left-handed, as determined by the Revised Edinburgh Handedness Inventory (Dragovic, 2004). All participants presented no neurological or neuromuscular disorders. The study was approved by the San Francisco State University Institutional Review Board committee.

### Apparatus

**Figure 1:**
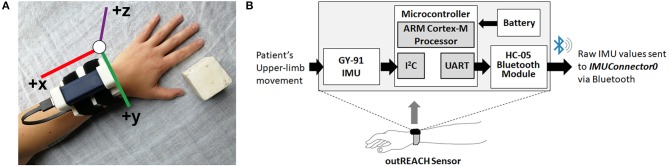
The outREACH sensor **(A)** and device placement on a participant's left arm and **(B)** schematic.

Data was collected from a custom-built wearable sensor (i.e., the outREACH sensor, Figure 1) that is 450 g in weight (sensor body = 83 × 59 × 39 mm, battery = 29 × 21 × 85 mm). The outREACH sensor was developed using low-cost, easy-to-assemble commercial components with a total cost of around $30 USD. The sensor consists of a Tiva C Series TM4C123G microcontroller featuring the ARM Cortex-M4 architecture (Texas Instruments), GY-91 MPU-9250 Sensor Module, HC-05 Bluetooth module, and a 2,600 mAh USB portable battery (Mophie). The GY-91 was selected for its low-cost (~10 USD), small size (14 × 21 mm), and fully integrated 10 degree of freedom measurement capabilities. The MPU-9250 sampling rate was set at 100 Hz, with the accelerometer and gyroscope configured for a range of ±4 g and ±500°/s, respectively. When the Tiva microcontroller is connected to the portable battery or reset, it automatically configures and calibrates the GY-91.

Initial calibration of the GY-91 occurs at the start of each data collection, after which point the raw accelerometer and gyroscope data are sent from the GY-91 via the Inter-Integrated Circuit Bus (I2C) communication protocol, over the HC-05 Bluetooth module via UART, to a custom PC recording software (IMUConnector0). Synchronization of the Vicon and outREACH sensor data was achieved via the third-party open source Auto Hotkey software, which was used to move the cursor to the specified positions on the computer screen and click on the Vicon Nexus and the IMUConnector0 buttons simultaneously to start data recording from both devices.

In addition, kinematic data were recorded using an eight-camera optical motion capture system (Bonita 10, VICON Motion Systems), with a temporal and spatial resolution of 200 Hz and 1 mm, respectively. Each trial was recorded using a Bonita 720c HD digital video camera that was placed above the apparatus and provide a bird's eye view of the apparatus and the participant. The digital video camera was synchronized with the Vicon motion capture system and was used to record initial grasp postures and identify any movement errors. The 3D coordinates of the retro reflective markers collected using the Vicon system were reconstructed and labeled for each individual trial in Vicon Nexus software (v2.2). Any missing data (<10 frames) were interpolated using a cubic spline, and processed in MATLAB using a custom program (The MathWorks, Version R2012a).

### Procedure

After reading and filling out the written informed consent and handedness inventory forms, the participant sat upright on a chair with a firm back and no armrests. A table was placed in front of them at a distance of 15 cm from anterior torso and at mid-abdomen height. The sensor was then placed on the tested wrist of the participant, and they placed their hand on the starting position located on the table in a pronated orientation. Upon the verbal signal from the experimenter, the participant performed the specified action, after which they returned the hand to the starting position. For all trials, participants were instructed to perform the movements at a comfortable speed and to grasp the object so that it does not slip through their fingers during transport. Trials performed in a manner that did not coincide with the instructions (moving prior to verbal start command, placing the object to the wrong target) were repeated immediately.

**Figure 2:**
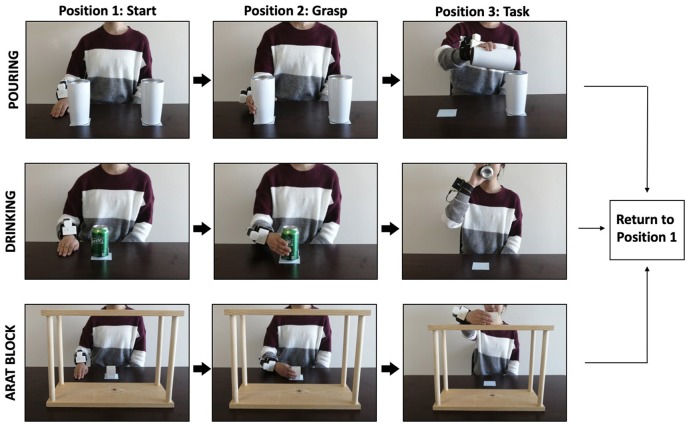
Example of a participant executing the pour water **(top panel)**, drink **(middle panel)**, and block task **(bottom panel)** while wearing the outREACH sensor.

Kinematic analysis was evaluated through the performance of three tasks commonly used to evaluate post-stroke upper limb function (Figure 2). The *Block* task is from the grasp subtest of the Action Research Arm Test (ARAT, Lyle, 1981) and requires the participant grasp a 5 cm^3^ block from the table, place it on the top of a 37-cm-high shelf placed 25 cm away from the proximal edge of the table, and then bring their hand back to rest on the table. The *Drink* task is from the grip subtest of the Frenchay Arm Test (FAT, Heller et al., 1987), and requires participants to pick up a water glass, pantomime drinking, place it back on the table, and then bring their hand back to rest on the table. The *Pour Water* task is from the grip subtest of the ARAT, and requires participants reach for a cup filled with water, pour the water into an empty cup, place the cup back on the table, and then bring their hand back to rest on the table.

The order in which the tasks were performed, and the hand used to perform each task, were blocked and counterbalanced across the participants. Participants performed each task 30 times with each hand (dominant, non-dominant), yielding a total of 60 trials. Between the six blocks, participants were given a 2-min rest period. The entire experiment lasted approximately 30 min.

### Data Processing

#### Vicon Module

The 3D coordinates of the Vicon markers were reconstructed and labeled for each individual trial, filtered using a Woltring filter with a predicted mean square error value of 5 mm^2^ (Vicon Nexus 2.2), and exported in CSV format. Using a custom written MATLAB (The MathWorks®, Version R2019a) script, the data was trimmed to coincide with the onset and offset of the raw IMU signal. The 3D position data of each axis were transformed into movement velocity using a first-order central difference technique.

#### outREACH Sensor Module

The raw IMU data was first trimmed to exclude data before 100 frames to the first significant movement (i.e., gyroscope velocity > 10°/s, z-axis acceleration >0.7 g, or absolute value of resultant velocity >0.1) and 100 frames after the last significant movement (i.e., gyroscope velocity > 5°/s, z-axis acceleration >0.7 g, or absolute value of resultant velocity >0.0025). The smaller threshold at the end of the trial was selected due to increased relative difficulty in ensuring the sensor was still (i.e., from vibrations associated with setting the sensor on the table). The resultant acceleration of the trimmed IMU data was calculated by passing the resultant acceleration through a high pass Butterworth filter (0.001 Hz), and then passing the absolute value of the high pass filter output through a 2 Hz low pass Butterworth filter in order to determine time periods where the sensor is stationary (Xio Technologies, 2017).

The IMU data was then split into start, center, and end sections. The start section contains data >10 frames from the start of the trimmed movement (i.e., stationary frames) to the first frame in which angular velocity >10°/s, and is calculated by determining the first 10 frames that are greater than threshold. The end section contains data >20 frames from the start of the trimmed signal to the last frame in which angular velocity >10°/s, and is calculated by determining the last 20 frames are greater than threshold. Only the remaining data (i.e., the center section) is used for further processing. An attitude heading reference system (AHRS) filter was used to compute current IMU orientation and to transform the data from the sensor frame to the earth frame (Madgwick et al., 2011), with 1 g subtracted from the z-axis acceleration to remove gravitational acceleration effects. Velocity was calculated by taking the integral of the acceleration signals up until a stationary period (where velocity = 0), with draft subsequently subtracted during non-stationary periods.

#### Data Analysis Module

The Vicon and IMU time series were cropped so that the remaining time period was between the moment when the hand left the starting position (movement onset) to the time the hand returned to the starting position (movement offset) was analyzed separately for each trial. Because of the variability in movement kinematics between tasks and participants, the MATLAB peak detect function was used to determine the expected number of peaks for each task (block = 3, drink = 3, water = 4) by using a minimum possible threshold of 0.1 m/s as the initial value, and then increasing the threshold by a factor of 1.05 for a maximum of 60 iterations. In the rare case that the peak detect function failed to find the expected number of peaks, the threshold was chosen based on the maximum threshold with the same number of peaks detected by both systems. Movement onset was determined as the instant when resultant velocity exceeded a given value (2% for Vicon, 1% for IMU) of the first velocity peak, and the leading 50 frames exhibiting a mean value <0.05 m/s. Movement offset was determined as the moment when resultant velocity dropped below 2% of the last peak, and the following 75 frames exhibiting a mean value <0.05 m/s.

### Statistical Analysis

Based on prior literature (cf. Nordin et al., 2014; Lee et al., 2015), three kinematic variables known for their high sensitivity to detect differences in upper limb motor dysfunction were selected: total movement time, peak velocity, and spectral arc length. Total movement time is defined as the time period from movement onset to movement offset. Peak velocity is defined as the highest point on the resultant velocity curve. Spectral arc length is a dimensionless measure of the arc length of the Fourier magnitude spectrum of the velocity signal (see Balasubramanian et al., 2011 for more details).

To validate the ability of the custom IMU to measure upper limb kinematics, values were compared against those computed by a Vicon motion capture system. Pearson product moment correlation coefficients (*r*) were calculated in order to quantify the degree to which Vicon and the IMU are related, with numerical values ranging from −1.0 (strongly negatively correlated) to +1.0 (strongly positively correlated). Inter-sensor reliability was assessed using intra class correlation coefficients (ICC_2, 1_) using the absolute agreement definition between the Vicon motion capture system and the IMU (Kim, 2013). The strength of the relationship was determined using Evans (1996) empirical classifications, in which a value lower than 0.20 was very weak, 0.20–0.39 was weak, 0.40–0.59 was moderate, 0.60–0.79 was strong, and 0.80–1.0 was very strong. In addition to the ICC, the levels of agreement between the two systems was calculated using Bland-Altman plots, separately for each variable (Bland and Altman, 1986). To this end, differences between the two systems was plotted against the mean of the two devices, thereby providing an indication of potential systemic bias between the devices.

## Results

Overall, 4,860 object manipulation trials were obtained with both the outREACH sensor and an 8-camera Vicon motion capture system (corresponding to 31 participants × 3 tasks × 2 hands × 10 trials). The outREACH sensor produced tracings representative of upper limb movements, with kinematics that did not differ from the reference system (see Figure 1). As can be seen in Table 1, almost perfect agreement was found between the outREACH sensor and Vicon system for all parameters and tasks (*r* = 0.808–0.990, ICC = 0.891–0.995), according to the benchmarks suggested by Evans (1996), with representative velocity trajectories for the two systems shown in Figure 2. When comparing the tasks, Pearson product moment correlation values (*r*) were highest for the block task (*r* = 0.977–0.989) than both the drink glass (*r* = 0.876–0.990) and pour water tasks (*r* = 0.808–0.977).

**Table 1:** Mean movement time, spectral arc length, and peak velocity values for the outREACH sensor and Vicon, with associated correlations and intraclass correlations between the two devices.

	**Vicon**	**IMU**	***r***	**ICC**
**ARAT block**				
Movement time	2,891.38	2,874.98	0.989^*^	0.973 (0.969–0.977)^*^
Spectral arc length	−2.348	−2.353	0.977^*^	0.989 (0.987–0.990)^*^
Peak velocity	1,204	1,184	0.980^*^	0.980 (0.976–0.988)^*^
**Drink glass**				
Movement time	4,275.71	4,281.71	0.876^*^	0.995 (0.991–0.996)^*^
Spectral arc length	−2.669	−2.742	0.990^*^	0.931 (0.919–0.941)^*^
Peak velocity	853	768	0.915^*^	0.954 (0.946–0.961)^*^
**Pour water**				
Movement time	4,704.15	4,715.01	0.977^*^	0.988 (0.986–0.990)^*^
Spectral arc length	−2.892	−2.789	0.914^*^	0.950 (0.942–0.957)^*^
Peak velocity	768	723	0.808^*^	0.891 (0.872–0.907)^*^

* *p < 0.05*.

**Figure 3:**
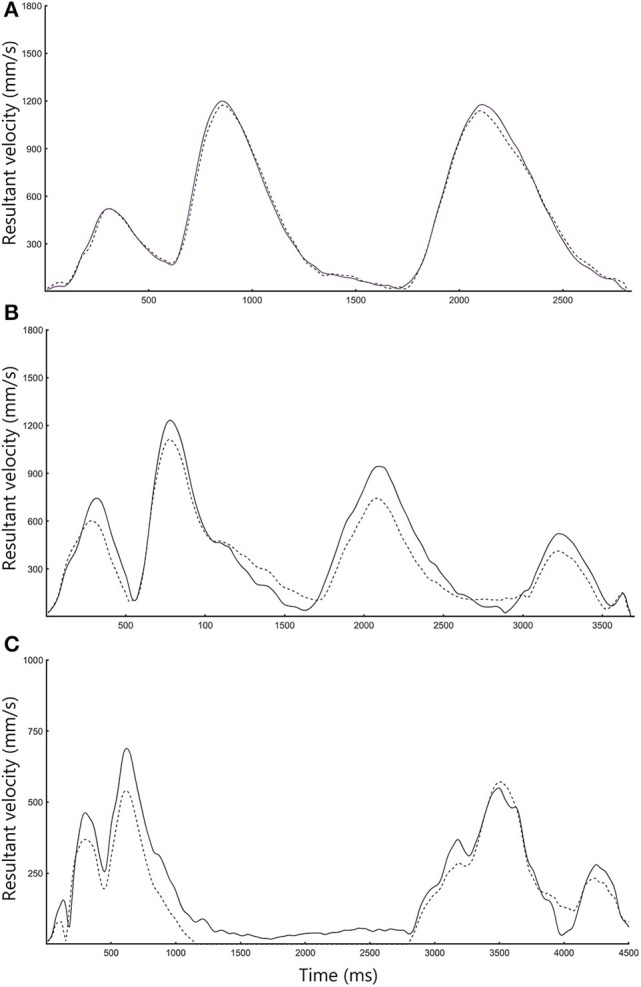
Representative velocity trajectories for the **(A)** ARAT Block, **(B)** Drink, and **(C)** Pour Water task. Black lines refer to Vicon data, whereas dotted lines refer to outREACH IMU data.

**Figure 4:**
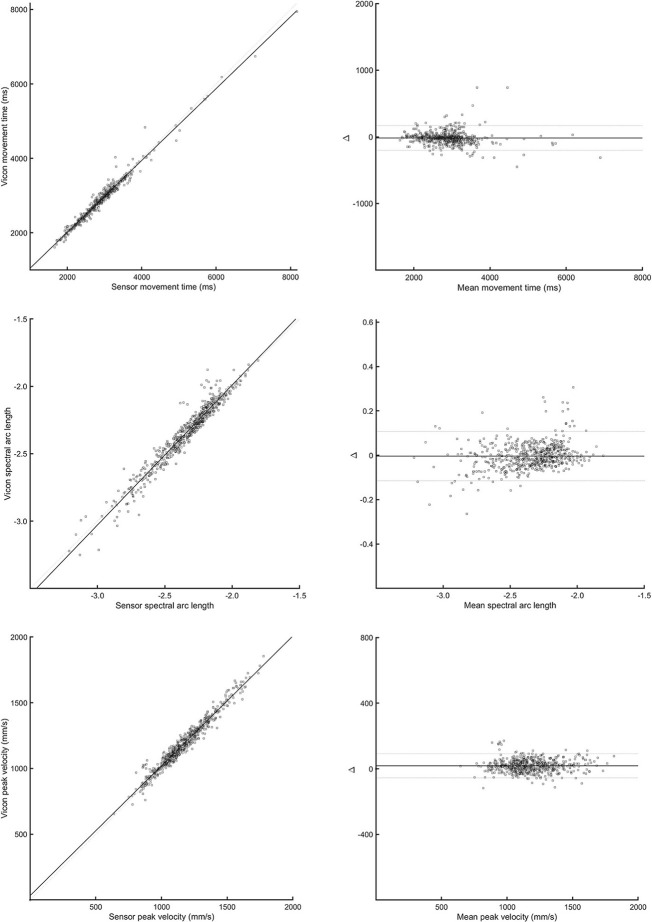
Correlation **(left panels)** and Bland-Altman plots **(right panels)** between the outREACH sensor and Vicon for the Block task. The top-most panels refer to differences in movement time values, middle panels refer to differences in spectral arc length values, and the lower-most panels refer to differences in peak velocity values.

Bland-Altman plots for each parameter and task (see Figure 3) indicated a high level of agreement between the outREACH sensor and the reference system. Limits of agreement (LOA, see Table 2) indicated that 95% of the differences in movement time measurements between the outREACH sensor and the reference system (Vicon) would be −200 to 170 ms for the Block task, −220 to 230 ms for the Drink Glass task, and −290 to 310 ms for the Pour Water task. LOA for spectral arc length would be −0.11 to 0.11 for the Block task, −0.53 to 0.38 for the Drink Glass task, and −0.32 to 0.53 for the Pour Water task. LOA for peak velocity would be −55 to 92 m/s for the Block task, −220 to 51 m/s for the Drink Glass task, and −230 to 140 m/s for the Pour Water task. An example of measurements from the two devices for the Block task is shown in Figure 4.

**Table 2:** Measurement differences between the outREACH sensor and Vicon systems.

	**Coefficient of variation %**	**Mean difference**	**Lower limit of agreement**	**Upper limit of agreement**
**ARAT block**				
Movement time	3.30	−16.00	−200.00	170.00
Spectral arc length	−2.40	0.00	−0.11	0.11
Peak velocity	3.20	18.00	−55.00	92.00
**Drink glass**				
Movement time	2.70	5.00	−220.00	230.00
Spectral arc length	−8.50	−0.07	−0.53	0.38
Peak velocity	8.40	−82.00	−220.00	51.00
**Pour water**				
Movement time	3.20	11.00	−290.00	310.00
Spectral arc length	−7.70	0.10	−0.32	0.53
Peak velocity	13.00	−45.00	−230.00	140.00

## Discussion

Optoelectronic motion capture systems (e.g., Vicon, Optotrak) are the most valid technology currently used to measure kinematics (e.g., movement smoothness, velocity) in stroke patients with motor dysfunction (Corazza et al., 2010), and as such are considered the “gold standard” for evaluating upper limb movements. Despite their high spatial and temporal sensitivity, their high cost, large space requirements, lengthy training and setup time, and technical knowledge requirements hamper the use of motion capture systems in resource-constrained environments settings. An alternative is to capture upper limb movement kinematics using wearable sensors. However, before such technology can be deployed outside of the laboratory setting, its performance against a gold standard must first be characterized.

In this paper, we introduced a new low-cost wearable sensor designed to enhance stroke evaluation and rehabilitation in sub-Saharan Africa and described validation of its performance against a reference optical motion capture system. Results indicated very high correlations (*r* range: 0.808–0.990) and agreement values (mean difference range: −1.60 to 1.10) with the reference system, regardless of task or kinematic parameter. Bland-Altman analyses indicated that average IMU accuracy of the movement time parameter was within 1.60 ms of the reference system, accuracy of IMU spectral arc length was within 0.10 units of the reference system, and peak velocity was within 0.8 m/s. Moreover, the line of equity was within the 95% of the mean difference, indicating that there were no significant systematic errors present.

The results of the present study are consistent with prior work demonstrating that wearable sensor systems are capable of accurately measuring upper limb joint angles (Peppoloni et al., 2013; Robert-Lachaine et al., 2017), joint range of motion (Lee et al., 2010; Timmermans et al., 2010), and limb usage (Uswatte et al., 2006). Although these studies have made valuable contributions to this line of work, most upper limb IMU systems are comprised of multiple sensors (e.g., 3 in Peppoloni et al., 2013; 17 in Robert-Lachaine et al., 2017) in order to reduce the large systematic errors (e.g., biases, drifts) often observed when using a single IMU. In contrast, we were able to ameliorate these issues by including a stationary detection function and complementary AHRS filter to the algorithm. In addition, to exhibiting similar performance values compared to the aforementioned work, the benefit of this approach is that the algorithm is less computationally complex than if multiple sensors are used, and the overall cost of the device is reduced.

The outREACH sensor described in this paper is well suited as a technological neurorehabilitation tool, as it is lightweight and can be worn directly on the user's wrist, thus minimizing intrusiveness and interference during the performance of activities of daily living. In addition, to improve uptake and scalability of the outREACH sensor for tele-rehabilitation purposes, we have considered the barriers to, and enablers of, stroke tele-rehabilitation from the perspective of sub-Saharan African healthcare professionals and stroke patients (cf. Hughes et al., 2018, 2019a,b). For example, in Hughes et al. (2019b), a user-centered approach was used to determine possible features that US- and Ethiopia-based rehabilitation clinicians felt should be implemented into the outREACH system. Results of that study indicated that Ethiopian clinicians felt that integrated sensors that collect quantitative data about movement quality and strategy would be a very important feature and should be included in a stroke tele-rehabilitation system. As this project moves forward, efforts will be focused on integrating the sensor into the outREACH mHealth application that leverages tele-monitoring and tele-consultation in order to improve rehabilitation access and care in sub-Saharan Africa stroke patients (Hughes et al., 2018, 2019a,b).

In addition to these efforts, we are cognizant that the single unit cost of the outREACH sensor (~$30 USD), although much lower than the commercial systems used in prior work [Xsens (Carpinella et al., 2014; van Meulen et al., 2015; Robert-Lachaine et al., 2017), Opal (Morrow et al., 2017)], is likely to be too expensive for the 33.5% of the Ethiopian population living below the international poverty line (US$1.90/day) (World Bank Group, 2015). As such, to ensure the long-term sustainability and scalability of the outreach sensor we are implementing best practices of mobile health (mHealth) projects that have successfully scaled-up and been sustained (cf. Aranda-Jan et al., 2014). Moreover, we are developing a social entrepreneurial subscription-based business model in which US- and African-based for-profit health facilities would pay a recurring fee to access the outREACH system, with the derived revenue used to provide the outREACH system to resource constrained clinics and persons at a substantially reduced cost.

While the current study makes an important contribution to this field of research, the high costs of optoelectric motion capture systems render them cost prohibitive for many sub-Saharan clinics and universities. As such, we were unable to conduct the validation study in the Ethiopia stroke population, but are currently to investigating the ability of the outREACH sensor to objectively quantify motor performance in Ethiopian stroke patients, as well as evaluate whether the sensor can discriminate between stroke patients with different impairment levels. As this line of work continues, we will work to ensure that online data processing can be achieved on the sensor or mobile phone rather than using processing the data offline using MATLAB software. In addition, given our strong interest in humanitarian engineering, we intend to reduce the weight, size, and cost of the sensor, and to make the described technology freely available and open-source to low-income countries, as well as those individuals who live in medically underserved areas of developed nations.

## Conclusion

The outREACH sensor utilized in this study demonstrated a high level of agreement with the gold standard technology (Vicon) in measuring upper limb movement kinematics, with almost perfect agreement found between the outREACH sensor and Vicon system for all parameters and tasks. Considering the convenience, ease of use, and cost of the sensor, it has the potential to provide clinicians in resource constrained areas, such as sub-Saharan Africa, with useful quantitative information that can help guide clinician reasoning.

## Data Availability Statement

The datasets generated for this study are available on request to the corresponding author.

## Ethics Statement

The studies involving human participants were reviewed and approved by San Francisco State University Institutional Review Board. The patients/participants provided their written informed consent to participate in this study.

## Author Contributions

CH, XZ, and AL designed the experiment and formulated the experimental question. XZ, AL, and SS developed the sensor. CH, AL, SS, and CG-M performed the data analysis and statistics. CH, CG-M, XZ, MB, GB, and AL wrote the paper. SS revised the final version of the manuscript.

### Conflict of Interest

The authors declare that the research was conducted in the absence of any commercial or financial relationships that could be construed as a potential conflict of interest.
